# The Relationship between Orthorexia Nervosa and Obsessive Compulsive Disorder

**DOI:** 10.3390/ejihpe13050065

**Published:** 2023-05-17

**Authors:** Mirko Duradoni, Mustafa Can Gursesli, Maria Fiorenza, Andrea Guazzini

**Affiliations:** 1 Department of Education, Literatures, Intercultural Studies, Languages and Psychology, University of Florence, 50135 Firenze, Italy; 2 Department of Information Engineering, University of Florence, 50139 Firenze, Italy; 3 Centre for the Study of Complex Dynamics, University of Florence, 50135 Firenze, Italy

**Keywords:** orthorexia nervosa, obsessive compulsive disorder, eating behavior, obsession, healthy foods

## Abstract

Orthorexia nervosa (ON) is characterized by an intense avoidance of foods considered unhealthy, obsession with healthy eating behaviors, and pathological fixation on healthy foods. Although there are still debates in the literature about the psychological factors and symptoms of ON, it should be noted that many of the symptoms share common features with obsessive compulsive disorder (OCD). The aim of the present study was to investigate the relationship between ON and OCD with its subtypes. In this framework, the cross-sectional study was conducted with an opportunistic sample of 587 participants (86% women and 14% men), with an average age of 29.32 (s.d. = 11.29; age range = 15–74). Our work showed that almost all OCD subtypes were largely correlated with ON. The lowest correlation was for “Checking” and the highest for “Obsession”. Overall, the OCD subtypes (i.e., Indecisiveness, Just Right, Obsession, and Hoarding) were more strongly associated with ON measures, while subtypes Checking and Contamination, although positively associated, had lower correlation coefficients.

## 1. Introduction

The expression “Orthorexia Nervosa” (ON) was first introduced in 1997 in an article published in the Yoga Journal by Steven Bratman [1]. The scientific literature, for a long time, has been largely absent on the topic, with only a first article in 2004 appearing in a peer-reviewed journal that described ON as a “maniacal obsession” in the pursuit of healthy foods [2]. In general, ON, refers to an excessive concern for healthy eating that involves avoidance of food products rated as “unhealthy”, an excessive amount of time spent acquiring information about food composition, and preparing specific foods based on criteria perceived as healthy [3,4]. Although this description might compare ON with anorexia nervosa (AN), these diagnoses differ in the fact that while people with AN are driven primarily by body image distortion, people with ON are guided by the desire for healthy eating [5,6]. In fact, as conceptualized by Donini and colleagues [2], in ON, the purity of food is valued above anything else, including deleterious health effects (e.g., an extremely restrictive diet). Researchers suggested that people with ON are anxious about not eating healthy, compulsively plan and prepare healthy meals, and feel superior to others when it comes to choosing healthy foods [2]. People with ON are distinguished by unyielding self-discipline, perceiving a sense of superiority, manifesting a teaching attitude toward those who do not pursue a healthy diet, and investing a considerable amount of time in the selection, buying, and preparation of specific healthy meals [2].

The main physical and psychological harms associated with ON are the compulsive behaviors aimed at protecting the “purity” of the diet, which can develop into pervasive and invasive behaviors that compromise the quality of life [7]. The feeling of having control over food is perceived as satisfying and reinforces this behavior, which can affect health status (e.g., deficiency of certain nutrients or malnutrition) as well as social and relationship life, manifested primarily in social isolation [1,8].

Currently, ON does not appear as a disorder associated with a specific diagnostic category; however, it is mentioned in the category “other” of “Feeding and Eating Disorders (i.e., anorexia nervosa, bulimia nervosa, binge eating disorders, etc.)” of the Diagnostic and Statistical Manual of Mental Disorders, Fifth Edition (DSM-5) [9]. Nevertheless, many researchers proposed different diagnostic criteria [10,11]: (a) obsessive behaviors and concern with healthy nutrition that includes a restrictive “healthy” diet (that the individual believes to be healthy and pure) and avoidance of foods believed to be unhealthy; (b) extreme emotional distress (i.e., feelings of guilt, shame, and/or anxiety) after violations of their restrictive dietary rules; (c) physical impairments (i.e., weight loss, malnutrition, and/or physical health complications); and (d) psychosocial impairments in social, vocational, and/or academic functioning. 

Obsessive compulsive disorder (OCD) refers to a mental condition where individuals grapple with intrusive thoughts and resort to rituals to alleviate their distress [12,13]. It is more accurate to classify that mental condition as part of the obsessive compulsive spectrum, which encompasses persistent mental or behavioral activities that consume a substantial amount of individuals’ time, with the intention of neutralizing invasive thoughts [13]. These activities can be defined as a set of behaviors that the person performs out of his/her will and sense of self by feeling compulsion. In this context, OCD can be explained as a disorder with two main contents: “Obsessions and Compulsions”. Obsessions are recurring thought patterns that do not leave the mind, which the person wants to get rid of but cannot get rid of. These thoughts can cause the person to experience anxiety due to the disturbing ideas they drive the person to. Compulsions (rituals) is the name given to all the mental or physical actions that the person performs at the end of obsessive thoughts by feeling obliged [14]. The person usually feels short-term relief when performing this behavior, but this feeling of relief is short-lived and the recurrent obsession that follows pushes the person to perform more compulsive behaviors.

In the previous paragraphs, many of the features described evoke similarities between OCD and ON [15]. This overlap with OCD appears in orthorexic people who manifest (a) recurrent and intrusive thoughts in relation to food and health; (b) excessive worry about contamination and impurity content; (c) ritualistic behaviors over food preparation and consumption; and mood changes based on successful adherence to self-imposed rules [4,16,17]. Indeed, orthorexic symptoms seem to be widely present in obsessive compulsive disorder patients [18,19]. 

One thing that instead appears to be different between ON and OCD is that the content of intrusive thoughts in ON is usually perceived as egosyntonic [20,21], in contrast to OCD, in which the beliefs associated with obsessions and compulsions are more often experienced as egodystonic [20,22]. 

The relationship between ON and other psychological disorders has been explored in several studies. Awad et al. found a positive correlation between ON and depression and anxiety, with a significant gender difference. Specifically, high anxiety was significantly associated with higher unhealthy orthorexic tendencies in women [23]. Rudolph’s study also found a positive correlation between ON and exercise addiction, with a higher correlation coefficient observed among female participants [24]. In addition, Barnes et al. conducted a correlation analysis and reported a significant positive association between elevated orthorexic tendencies and various dimensions of perfectionism, including self-oriented, others-oriented, and socially prescribed aspects, as well as appearance orientation, preoccupation with being overweight, self-reported weight, and fearful and dismissing attachment styles. Moreover, higher levels of orthorexia were negatively correlated with body satisfaction and a secure attachment style [25].

Several studies already available in the literature reveal that ON and OCD have yielded different results and these diagnoses have been examined differently [16,21,26,27]. However, the research in the literature has focused only on the general structure of OCD (or several subgroups), neglecting the sub-categories of OCD [16,18,19,28]. This makes it difficult to study a complex disorder such as OCD with specific subgroups and also reduces the specificity and predictiveness of the results. The conducted study aims to examine the relationship between ON and obsessive compulsive disorder (OCD). Thus, in this study, we tried to obtain a relevant result without neglecting the subgroups of ON and OCD.

## 2. Materials and Methods

### 2.1. Measures

Information on sex and age was collected from the participants through a demographic information form.

The Italian validation of the Vancouver Obsessional Compulsive Inventory (VOCI) was used to measure participants’ cognitive and behavioral OCD components [29,30]. It contains a total of 55 items measured on a 5-point Likert scale and divided into 6 different subscales defined in order: Checking (α = 0.96), Contamination (α = 0.92), Hoarding (α = 0.92), Obsession (α = 0.88), Just Right (α = 0.89), and Indecisiveness (α = 0.85). “Checking” OCD subtype entails performing safety checks to prevent obsessive thoughts and reduce uncertainty. Obsessions and compulsions associated with “contamination” OCD type revolve around both plausible and implausible contagions or contaminations. Hoarding, which is a prevalent subtype of subclinical OCD, is marked by the excessive accumulation of objects and the inability to discard them. Obsession is a form of OCD that individuals cannot get rid of and cannot stop thinking about. The “Just Right” OCD subtype is identified by an inner drive to alleviate discomfort by performing specific actions to achieve a sense of satisfaction that everything is “just right”. The concept of “indecisiveness” is characterized by prolonged decision-making times and heightened information-seeking behaviors, and research indicates a link between this trait and the presence of OCD symptoms and tendencies. The items of the subscales showed adequate internal consistency, and the overall Cronbach’s α was 0.94.

ORTO-R [31], which is a revised version of ORTO-15, contains six items and is evaluated on a four-point Likert scale. In the conducted study, it was used to measure the ON behaviors of the participants and Cronbach’s α was 0.75.

Orthorexia Nervosa Inventory (ONI) is a 4-point Likert scale consisting of 24 different statements, measuring the eating behaviors of individuals in 3 different factors [32]. Cronbach’s α was 0.94 overall, and ONI demonstrated good internal consistency with the other three factors Impairments (α = 0.90), Behaviors (α = 0.89), and Emotions (α = 0.88).

In order to increase the strength of our research design with respect to the ON construct, we used two important measures found in the literature that vary based on dimensionality (one multidimensional and one monodimensional) and recency (i.e., publication date). This approach allowed us to address current variations in the operationalization of ON.

### 2.2. Sample and Sampling

To determine the appropriate sample size for our study, we performed a power analysis using G*Power [33,34]. Because the authors planned to use Pearson correlations to examine the relationship between OCD and ON scores, a power analysis was calculated for this type of analysis. The power analysis indicated that a sample size of 150 participants would be required to achieve a statistical power of 0.80 while capturing a typical effect size (r = 0.20), assuming a significance level of 0.05. In addition, we considered the sample size required to achieve a stable measurement-error-free correlation. In our case (i.e., population correlation q = 0.20; composite score reliability derived from other works ω = 0.80), a stable measurement-error-free correlation would be met at 360 [35]. Power analysis with respect to sex differences indicated that a sample size of 204 would be sufficient to ensure the same statistical power (i.e., 0.80) and effect size (d = 0.50), even when considering an allocation ratio N2/N1 of 6. Because the number of participants recruited for the study was 587, we considered our sample size to be sufficient for our research purposes. Participation was encouraged through posts and messages on social media platforms such as Facebook and Instagram, as well as through direct solicitation by scanning a QR code that led to the online data collection form. Data were collected in compliance with Italian data protection regulations (Legislative Decree DL -101/2018) and EU regulations (2016/679). In the end, 587 people (86% female), with an average age of 29.32 (s.d. = 11.29; age range = 15–74), participated in and completed the survey.

### 2.3. Data Analysis

The conditions required for the inferential analyses were verified using the collected data. For all continuous variables studied, the normality of the distribution was assessed by analyzing the asymmetry and kurtosis values. Common method bias was also tested using Harman’s single-factor test. To test for sex differences, Welch’s t-test was used because it performed better than the Student’s t-test when sample size and variance were unequal between groups and gave the same result when sample size and variance were equal [36]. Finally, partial correlations were used to estimate the relationships between OCD and ON after controlling for participants’ sex.

## 3. Results

As a first step, we produced descriptive statistics for the collected variables. We used mean and standard deviation for continuous variables and percentages for discrete ones. As shown in Table 1, data were presented in a sex-sensitive way (i.e., disaggregated by sex where applicable). In addition, for each OCD type, the percentage of people over the 90° percentile threshold is indicated.

In our sample, Just Right, Indecisiveness, and Contamination appeared as the most prevalent OCD subtypes, while Checking, Hoarding, and Obsession were the least common. In line with the literature, participants’ sex appeared to affect most of the OCD dimensions (Welch’s t_(contamination)_ = −4.19; *p* < 0.001; Welch’s t_(Indecisiveness)_ = −3.08; *p* = 0.003; Welch’s t_(Just Right)_ = −2.33; *p* = 0.02; Welch’s t_(Obsession)_ = −2.73; *p* = 0.007) and orthorexia scores (Welch’s t_(ONI impairments)_ = −2.67; *p* = 0.008; Welch’s t_(ONI emotions)_ = −2.11; *p* = 0.04; Welch’s t_(ONI total)_ = −2.25; *p* = 0.03; Welch’s t_(ORTO-R)_ = −4.42; *p* < 0.001). For this reason, we controlled for sex as a possible confounding variable to test the relationship between OCD and ON through partial correlation (Table 2).

## 4. Discussion

Scientific contributions to ON have increased rapidly and exponentially in recent years. However, despite this wealth of literature, there is no remarkable clarity on ON. Indeed, many uncertainties exist regarding the etiology, predisposing factors, and assessment of ON [37]. Nevertheless, several studies [2,19,38,39,40] have highlighted the presence of many compulsive traits in orthorexic behavior. Therefore, the aim of this study was to investigate the relationship between ON and obsessive compulsive disorder (OCD). The reported results showed that OCD and its types were found to have typical to relatively large correlations with ON, with the lowest correlation in “Checking” and the highest correlation in “Obsession”. Overall, Indecisiveness, Just Right, Obsession, and Hoarding were the OCD subtypes that were more strongly associated with ON measures, while Checking and Contamination, despite being positively associated, had lower correlation coefficients. Among the ONI dimensions, Impairments resulted as the least associated with each OCD type, which was to be expected given that OCD is less associated with physical impairments than ON, which can instead severely affect physical health [32,41], whereas the emotional and behavioral dimensions of ON are consistently more strongly associated with the OCD score for each OCD type.

Overall, our work has helped shed light on the overlap between OCD and ON. Although some of the features of ON are similar to those of OCD, these conditions are theoretically and empirically distinct [16,42]. For instance, “Just Right”, an OCD subtype characterized by the uncomfortable feeling that something is not quite right, has some similarities to ON since orthorexic people typically think that the food being consumed or to be consumed might be not right/healthy [43,44]. However, “Hoarding”, which is an OCD type characterized by a tendency to collect and an inability to discard the collected items, does not fit very well with the features of ON [14,44]. The fact that the observed correlation was between typical and relatively large correlations supported the distinction between the two conditions, as even among the strongest associations, only around 12% appeared to be common between OCD and ON. Thus, suggesting that ON could not be regarded as an epiphenomenon of OCD.

Notwithstanding, there are several limitations that must be acknowledged in relation to this study. First, our results are correlational; therefore, no causal relationship between the variables can be inferred. Second, the results are based on a biased sample due to non-random sampling and self-selection bias. Therefore, the generalizability of our results may be limited. Third, there is currently no definitive evidence that these instruments used in the study can be considered totally valid for all age groups. Lastly, the nature of self-report measures could have produced certain measurement biases, primarily due to the commonly observed inclination of individuals to provide socially desirable responses rather than truthful ones, which can compromise the accuracy of the data. Additionally, people’s limited capacity to evaluate themselves accurately could have further contributed to the potential measurement biases [45,46].

The present study provides a novel perspective on the ongoing academic debate regarding the relationship between ON and OCD, particularly with regard to the subtypes of OCD. While the majority of the scientific community suggests that the effects of ON should be analyzed within the framework of “Feeding and Eating Disorders” [47], the symptomatic similarities between the subtypes of OCD and ON suggest that the discussion regarding the classification of ON as a separate diagnosis will continue. As well as its scientific contributions, this study has practical value for professionals seeking diagnostic support, particularly for conditions such as ON where diagnostic criteria remain unclear. While previous studies have investigated the effects of various therapy techniques on eating disorders and OCD [48,49], the resources available for therapeutic interventions in the case of ON remain scarce. Therefore, recent studies have become a crucial reference for practitioners, enabling them to recognize the parallels between ON and OCD and to address the disorder effectively.

Future research should take the limitations into account and extend the research on ON by examining possible psychological factors that may be associated with ON’s manifestation, such as mattering [50], need for control [51], and narcissism [52]. 

Indeed, the excessive and recursive preoccupation with healthy food and eating might be maintained for social reasons, such as being valued and validated within a community. In this sense, orthorexic behavior could be reinforced by external validation and social feedback, especially for people such as narcissists who need to maintain their grandiose and unrealistic positive self-image [53,54,55]. In addition, as with pro-ana and pro-mia groups, social polarization could occur [56,57] in people sharing orthorexic eating habits, exacerbating already quite restrictive and privative dietary choices and beliefs, leading to consequences such as social isolation from others who do not share the same dietary choices [58]. In this sense, the integration of orthorexic people within social groups other than the ones sharing the same choices and beliefs should be further explored. 

One potential avenue for future research involves examining the connection between orthorexia and cyberchondria, which refers to the excessive and repetitive searching for medical information online [59,60,61]. Both of these phenomena stem from heightened health concerns and have been linked to symptoms of obsessive compulsive disorder [62,63,64]. Additionally, considering that cyberchondria has been found to be strongly associated with health anxiety [61], it would be worthwhile to investigate the relationship between orthorexia and health anxiety as well. This exploration could shed light on whether both conditions may be considered as secondary manifestations of an ineffective coping strategy in response to health anxiety.

Furthermore, in conducting future studies on this issue, developing various strategies to enlighten the public about the potential risks associated with an emerging and harmful phenomenon such as ON is imperative [65,66]. Examples of possible strategies include, but are not limited to, social media campaigns [67,68,69], mobile applications [70], gamification/video games [71,72,73], and television programs [74,75].

## 5. Conclusions

Overall, by analyzing the relationship between OCD subtypes and ON, we were able to appreciate how some types of OCD (i.e., Indecisiveness, Just Right, and Obsession) are more strongly related to ON than others (i.e., Checking). Despite the overlap between OCD and ON, the magnitude of their relationship suggests that the two conditions should be kept distinct.

## Figures and Tables

**Table 1:** Descriptive statistics of the variables included in the data collection disaggregated by sex.

	Total Sample	Females	Males	OCD > 90p	OCD > 90 (Females)	OCD > 90 (Males)
	M (s.d)	M (s.d)	M (s.d)	%	%	%
OCD: Checking	3.38 (4.56)	3.41 (4.65)	2.98 (4.25)	15.3%	14.8%	17.1%
OCD: Contamination	7.95 (8.05)	8.26 (8.20)	5.06 (6.05)	25.7%	26.6%	13.4%
OCD: Hoarding	3.56 (4.59)	3.48 (4.57)	3.46 (4.30)	15.3%	15.0%	13.4%
OCD: Indecisiveness	6.07 (4.71)	6.25 (4.82)	4.73 (4.01)	24.7%	26.2%	14.6%
OCD: Just Right	9.28 (7.40)	9.47 (7.46)	7.61 (6.55)	27.1%	27.7%	19.5%
OCD: Obsession	5.68 (6.86)	5.85 (6.97)	4.00 (5.42)	12.4%	13.1%	4.9%
ONI impairments	14.40 (4.58)	14.54 (4.81)	13.51 (2.86)	-	-	-
ONI behavior	13.47 (4.09)	13.51 (4.24)	12.96 (3.00)	-	-	-
ONI emotions	7.25 (2.61)	7.30 (2.70)	6.82 (1.83)	-	-	-
ONI total	35.11 (10.75)	35.35 (11.24)	33.28 (6.95)	-	-	-
ORTO-R	10.94 (3.29)	11.10 (3.34)	9.72 (2.46)	-	-	-

Note: N = 587; M = mean; s.d. = standard deviation; OCD = obsessive compulsive disorder; ONI = Orthorexia Nervosa Inventory.

**Table 2:** Partial correlation of the relationships between OCD subtypes and ON dimensions controlled for participants’ sex.

	ONI Impairments	ONI Behavior	ONI Emotions	ONI Total	ORTO-R
OCD: Checking	0.16 ***	0.21 ***	0.19 ***	0.19 ***	0.19 ***
OCD: Contamination	0.15 ***	0.21 ***	0.18 ***	0.19 ***	0.24 ***
OCD: Hoarding	0.21 ***	0.23 ***	0.24 ***	0.24 ***	0.28 ***
OCD: Indecisiveness	0.26 ***	0.27 ***	0.31 ***	0.29 ***	0.32 ***
OCD: Just Right	0.27 ***	0.30 ***	0.32 ***	0.30 ***	0.34 ***
OCD: Obsession	0.30 ***	0.30 ***	0.35 ***	0.32 ***	0.35 ***

Note: N = 563; OCD = obsessive compulsive disorder; ONI = Orthorexia Nervosa Inventory; *** = *p* < 0.001.

## Data Availability

The data presented in this study are available on request from the corresponding author.
